# A novel polysaccharide hemostatic agent in prevention of post-procedural bleeding following large colonic polyp resection

**DOI:** 10.1055/a-2794-0465

**Published:** 2026-02-09

**Authors:** Francesco Auriemma, Gianluca Franchellucci, Gianluca Andrisani, Francesco Di Matteo, Luca De Luca, Diletta De Deo, Federica Calabrese, Matteo Fiacca, Francesco Minnini, Danilo Paduano, Carmine Gentile, Paola Petrillo, Daryl Ramai, Antonio Facciorusso, Alessandro Repici, Benedetto Mangiavillano

**Affiliations:** 1 Gastrointestinal Endoscopy Unit, Humanitas – Mater Domini, Castellanza (VA), Italy; 2 9268 Digestive Endoscopy Unit, IRCCS Humanitas Research Hospital, Rozzano, Italy; 3 437807 Department of Biomedical Sciences, Humanitas University, Pieve Emanuele, Milan, Italy; 4 220431 Digestive Endoscopy Unit, Campus Bio-Medico University Hospital, Roma, Italy; 5 444273 Endoscopic Unit, ASST Santi Paolo e Carlo, Milano, Italy; 6 1861 Division of Gastroenterology, Hepatology, and Endoscopy, Brigham and Women's Hospital, Boston, United States; 7 18976 Department of Experimental Medicine, Section of Gastroenterology, University of Salento, Lecce, Italy

**Keywords:** Endoscopy Lower GI Tract, Lower GI bleeding, Polyps / adenomas / ..., Endoscopic resection (polypectomy, ESD, EMRc, ...)

## Abstract

**Background and study aims:**

Preventing post-procedural bleeding (PPB) after endoscopic mucosal resection (EMR) and endoscopic submucosal dissection (ESD) is crucial for minimizing post-endoscopic complications. We aimed to evaluate the rate of PPB in large colorectal lesions removed via EMR or ESD, followed by application of a resorbable plant-based hemostatic powder (HaemoCer PLUS).

**Patients and methods:**

This prospective pilot study was conducted at three tertiary care centers from November 2021 to February 2024. HaemoCer PLUS was applied at the end of the procedure and spread over the post-resection surface. No endoscopic clips were used after resection.

**Results:**

The study included 50 patients with a mean lesion diameter of 52.28 mm (standard deviation 18.29 mm). Fifty-six percent of the polyps were in the rectum and 86% were classified as adenomas. ESD was used to remove 20 lesions, hybrid ESD for eight, Hot-EMR for 17, and cold-EMR for five. All patients received HaemoCer PLUS application for PPB prevention. Delayed bleeding was observed in 6% of cases, including one case of ESD and two cases of hot-snare resection. PPB occurred 24 hours after the procedure and no bleeding cases were reported more than 30 days post-endoscopy. Univariate analysis showed no statistically significant associations between post-procedural bleeding and lesion size, patient age, or endoscopic resection technique.

**Conclusions:**

Use of a novel resorbable plant-based hemostatic powder could be a beneficial method for reducing delayed bleeding complications, particularly in high-risk groups characterized by specific lesions and patient factors.

## Introduction

Endoscopy has seen significant advancements and widespread adoption over the last two decades 1 2 . This operative landscape has led to improvement and establishment of new endoscopic standards for management of gastrointestinal (GI) precancerous lesions 3 . However, growing adoption of endoscopic procedures has also led to an increased focus on minimizing post-endoscopic complications, with bleeding and perforations being the most concerning 1 2 .

Gastrointestinal bleeding associated with endoscopic procedures is defined by clinical evidence of bleeding and a hemoglobin drop ≥ 2 g/dL on the day of the procedure (early bleeding) or up to 28 days post-procedure (delayed bleeding) 2 4 . Gastrointestinal bleeding is a common complication of procedures such as endoscopic mucosal resection (EMR) and endoscopic submucosal dissection (ESD) 3 4 . To this end, several factors influence bleeding risk, including size and location of the lesion, endoscopic technique, and use of anticoagulants or antiplatelet therapy 5 6 .

Traditionally, endoscopic treatments for bleeding management have included injection therapy (e.g., epinephrine, sclerosing agents), mechanical therapy (e.g., hemostatic clip placement), and thermal therapy (e.g., monopolar and bipolar coagulation, argon plasma coagulation, or heater probe). For controlling post-EMR/ESD bleeding, current treatments include injection therapy, monopolar electrocoagulation, ligation with a detachable snare, and endoscopic clipping 7 8

Overall, post-procedural bleeding ranges between 5% to 10% for large EMR and ESD procedures, depending on data reported in the literature 1 9 10 11 12 . Post-procedural bleeding requires endoscopic assessment and contributes significantly to the operational burden of endoscopy services. To reduce post-procedural bleeding rates, new endoscopic hemostatic methods, including topical hemostatic agents, are emerging as potential alternatives for preventing bleeding or for treating bleeding refractory to standard therapies 13 14 . Today, several topical hemostatic agents are available, with different mechanisms of action. Starch-derived powders absorb the aqueous component of blood with ensuing concentration of clotting elements. Generally amylase enzymatically degrades these substances within 48 to 72 hours, with a limited risk of granuloma formation 15 . Another agent, TC-325, is a bentonite clay powder that possesses similar activity; however, it is non-absorbable and uses a CO _2_ -powered, non-contact delivery system 16 . Finally, polymer-based agents such as UI-EWD form a mucoadhesive hydrogel that lasts up to 48 hours and is applied via a controlled, layer-by-layer delivery method 17 .

The aim of this study was to assess efficacy and safety outcomes following application of a resorbable plant-based hemostatic powder (HaemoCer PLUS - BioCer Entwicklunga - GmBH) following resection of large colorectal polyps.

## Patients and materials

### Study design

This was a multicenter pilot prospective study conducted in three Italian tertiary centers. The enrollment period was from November 2021 to February 2024. The protocol was approved by the Institutional Review Board of Humanitas Mater Domini (no. 488/22). Informed consent was obtained prior to enrollment. Inclusion criteria were: 1) patients who authorized use and processing of their details for the purpose of research; 2) patients able to express consent; 3) age > 18 years; 4) EMR with hot or cold snare or ESD of lesions > 30 mm in diameter; and 5) procedures using HaemoCer PLUS for bleeding prevention. Exclusion criteria were: 1) hemostatic therapies other than HaemoCer PLUS; 2) clip closure; and 3) known coagulopathy likely to affect the risk of bleeding; and 4) anticoagulant and antiplatelet therapy assumption according to European Society of Gastrointestinal Endoscopy guidelines 18

### Study aim

The study aimed to assess the post-procedure bleeding rate for large colorectal lesions removed through EMR or ESD followed by application of a resorbable plant-based hemostatic powder (HaemoCer PLUS). Post-procedural bleeding was defined as bleeding occurring after the procedure or up to 30 days 2 . Severity of bleeding was classified according to the American Society for Gastrointestinal Endoscopy (ASGE) lexicon 19 . The secondary aim of the study was to evaluate safety of the powder based on adverse events (AEs).

### HaemoCer PLUS

HaemoCer PLUS is a resorbable plant-based hemostatic powder containing no animal or human components. Hemostasis occurs by rapidly accelerating the normal physiologic clotting cascade without use of chemical or pharmaceutical ingredients. Once in contact with blood, HaemoCer PLUS enhances the natural clotting cascade by rapidly dehydrating the blood and accelerating concentration of platelets, red blood cells, and coagulation proteins at the bleeding site. The second mode of action of HaemoCer PLUS is formation of a robust gelled matrix that adheres to the bleeding site and forms a mechanical barrier to prevent further bleeding. HaemoCer PLUS is also indicated for prevention of postoperative adhesions following surgical interventions in cavities with a mesothelial lining. HaemoCer PLUS is rapidly resorbed by amylase, typically within a few days, without leaving any residue behind, depending on the amount and the place of application.

### Procedure

Informed consent was obtained from all patients before they underwent endoscopic resection of colorectal lesions. In all cases, a forward-view endoscope was used, with the diameter varying according to endoscopist preference. The procedures were performed by an experienced endoscopist, skilled in both EMR and ESD, using carbon dioxide insufflation. All procedures were conducted in accordance with local policies and under deep sedation with propofol or general anesthesia administered by an anesthesiologist or anesthesiology assistant.

During the procedures, intraprocedural bleeding was recorded along with the methods used for bleeding control. Preemptive coagulation was performed on bleeding vessels encountered during ESD and on the scar tissue following resection using the EMR technique. HaemoCer PLUS was administered at the conclusion of the procedure and applied over the post-resection surface. No endoscopic clips were placed after resection or after HaemoCer application. Coagulation of vessels was performed only in cases of active bleeding during ESD or EMR.

The HaemoCer Endoscopic Applicator was used for these applications. The applicator features a tube length of 2400 mm, an outer tube diameter of 2.5 mm, and a pump ball serving as a pneumatic source. In addition, it includes a built-in HEPA (High-Efficiency Particulate Air) filter to purify ambient air. This applicator requires no additional handling devices (such as an air compressor) and is designed for simple and uncomplicated use.

### Statistical analysis

This study was designed as a preliminary investigation into use of HaemoCer PLUS for endoscopic resection; for this reason, a formal a priori power calculation was not performed, given lack of existing data. A pragmatic sample size of 50 patients was chosen to provide an initial assessment of agent safety, feasibility, and therapeutic efficacy *.* Descriptive analysis of quantitative data with normal distribution was expressed using means and standard deviations (SDs). Categorical variables were expressed with frequency and percentages. A normal distribution was verified using the Shapiro-Wilk test. Quantitative variables were compared using Student *t* -test, and categorical variables using chi-square or Fisher’s exact test. Logistic regression was used to explore significant correlations between predictive variables and outcomes. *P* ≤ 0.05 was considered statistically significant. Statistical modelling and tests were performed using STATA software version 18.0.

## Results

### Study population

Overall, 50 patients were enrolled, 62% of whom were male. Mean age of the cohort was 72.14 years (SD 11.40 years). Mean diameter of colonic polyps was 52.28 mm (SD 18.29 mm). Polyps were primarily located in the rectum (56%), followed by the ascending colon (20%), sigmoid colon (12%), transverse colon (8%), and cecum (4%) ( Table 1 ).

**Table:** **Table 1** Overall patient characteristics.

**Patients**	**50**
Technique	50
	20 ESD
	8 Hybrid-ESD
	17 Hot-EMR
	5 Cold-EMR
Age, y (SD)	72.14 (11.40)
ESD	69.8 (10.64)
Hybrid-ESD	72 (3.54)
Hot-EMR	73.18 (14.67)
Cold-EMR	78.2 (9.67)
Sex, male	31
ESD	12
Hybrid-ESD	5
Hot-EMR	12
Cold-EMR	2
Dimension, mm (SD)	52.28 (18.29)
ESD	58.7 (15.03)
Hybrid-ESD	56.8 (17.91)
Hot-EMR	47.64 (20.85)
Cold-EMR	35 (3.53)
Location	28 rectal
	6 sigmoid colon
	4 transverse colon
	10 ascending colon
	2 cecum (ileocecal valve included)
LST classification	13 LST-G
	21 LST-GM single nodule
	10 LST-GM multiple nodules
	6 LST-NG
Pathology	4 adenoma tubular low-grade dysplasia
	13 adenoma tubular high-grade dysplasia
	9 adenoma tubulovillous low-grade dysplasia
	17 adenoma tubulovillous high-grade dysplasia
	6 superficial invasive cancer
	1 hyperplastic
Intraprocedural bleeding	44 none
	3 not requiring additional devices (other than knife or snare tip coag)
	3 requiring hot forceps
Final hemostasis	15 no hemostasis
	33 snare tip or knife soft coag
	2 forced coag
Postprocedural bleeding	
Overall	3
ESD	1
Hybrid-ESD	0
Hot-EMR	2
Cold-EMR	0
Bleeding complication according to ASGE lexicon	1 mild
	2 moderate
Anti-platelet therapy	36 none
	9 aspirin/NSAIDs
	2 ticagrelor
	2 picotamide
	1 indobufen
Anti-coagulant therapy	42 none
	2 apixaban
	2 dabigatran
	1 rivaroxaban
	3 LMW Heparin
Ongoing antiplatelet therapy	9
Ongoing anticoagulant therapy	1
Ongoing both anticoagulant and antiplatelet therapy	2
Comorbidities	1 renal impairment (eGFR: 45 mL/min)
	24 cardiovascular disease
	2 chronic liver disease
ASGE, American Society for Gastrointestinal Endoscopy; eGFR, estimated glomerular filtration rate; EMR, endoscopic mucosal resection; ESD, endoscopic submucosal dissection; G, granular; GM, granular mixed; LST, lateral spreading tumor; NG, nongranular; NSAID, nonsteroidal anti-inflammatory drug; SD, standard deviation.	

Most lesions were classified as adenomas (86%), whereas 13 polyps had low-grade dysplasia, 30 high-grade dysplasia, six invasive neoplasia (T1a), and one lesion was described as hyperplastic. Among the patients, 15 were undergoing antiplatelet therapy and eight were receiving anticoagulant treatment. Four of these patients were simultaneously on anticoagulant and antiplatelet therapies. Eight patients received antiplatelet therapy as primary prophylaxis. Among the eight patients receiving anticoagulant therapy, three received novel oral anticoagulants (NOAs) for permanent atrial fibrillation, two received NOAs for thromboembolism prevention, and three patients received low molecular weight (LMW) heparin as a bridge with K antagonist for prosthetic heart valve and/or portal vein thrombosis treatment.

### Endoscopic resection

Twenty lesions were removed using ESD, eight with hybrid ESD, 17 with hot-EMR, and five with cold-EMR. Intra-procedural bleeding occurred in six cases (12%), three of which were treated using snare tip coagulation and three with hot forceps. Prophylactic hemostasis of visible vessels during or at the end the procedure was performed using knife or snare tip soft coagulation in 33 cases and knife or snare tip forced coagulation in two cases. Fifteen cases received no prophylactic hemostasis of visible vessels.

### Post-procedural bleeding

**Fig. 1:**
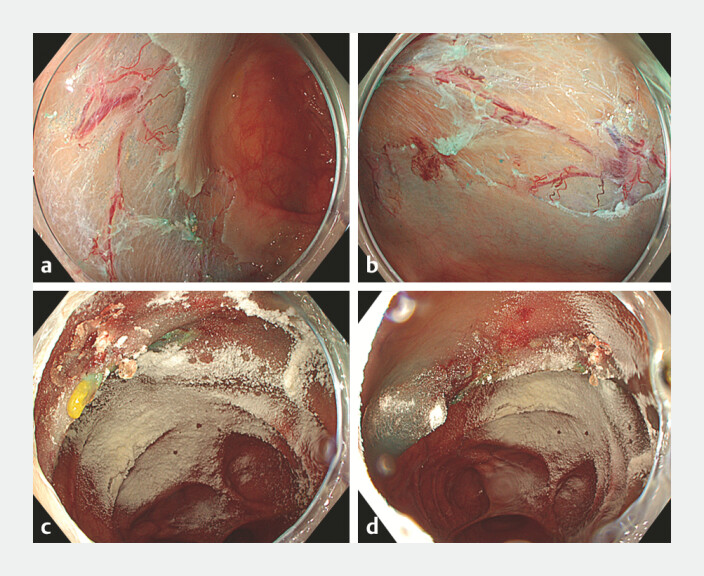
Haemocer application LST-G of ascending colon treated with underwater hot EMR. **a,b** Before application. **c,d** After application.

None of the lesions had active bleeding at the time of HaemoCer application ( Fig. 1 ). All patients received HaemoCer PLUS application for post-procedural bleeding prevention. From the study cohort, 47 lesions with a mean size of 51 mm (SD 18.97 mm) were administered a single dose of HaemoCer. Three larger lesions with a median size of 70 mm (interquartile range 67–80) were administered two doses of HaemoCer. During the follow-up period, there were three bleeding events (6%). All cases of bleeding were diagnosed 24 hours after the procedure. One bleeding event occurred in the ESD group (1/20, 5%) and two occurred in the hot-snare resection group (2/17, 11.76%). Post-procedural bleeding events in the study occurred in patients with no prior record of procedural bleeding.

Bleeding complications involved lesions located in the rectum (removed by ESD) and in the cecum (both removed by hot-snare). All the lesions were assessed as laterally spreading tumors-granular mixed (LST-GMs) with a single nodule and diameter > 20 mm. The rectal lesions treated by ESD were diagnosed as invasive cancer (T1a) by a pathologist whereas the cecal lesions were characterized by high-grade dysplasia with no invasive component detected. Both cecal bleeding events occurred in patients on single antiplatelet therapy with ticagrelor which could not be discontinued following cardiology consultation, and who were also on anticoagulant therapy with LMW heparin for permanent atrial fibrillation. These patients also had chronic liver disease and the last dose of LMW heparin was administered at least 24 hours before the procedure as per cardiology recommendations.

In univariate analysis, no statistically significant associations were found between post-procedural bleeding and lesion size, patient age, or endoscopic resection technique ( Table 2 ) No other complications (such as perforation, infection, or agent-related AEs) observed in the enrolled population.

**Table:** **Table 2** Univariate analysis.

**Characteristic**	**Odds ratio (95% CI)**	***P* value **
Sex	0.81 (0.069–9.54)	0.85
Age (years), mean (SD)	0.95 (0.87–1.04)	0.35
Dimension (mm), mean (SD)	1.025 (0.97 1.08)	0.97
Technique, n (%)		
Hot-EMR	1.00 (Reference)	-
ESD	2.533 (0.21- 30.69)	0.465
CI, confidence interval; EMR, endoscopic mucosal resection; ESD, endoscopic submucosal dissection; SD, standard deviation.		

## Discussion

Post-procedural bleeding is a significant complication that can occur after endoscopic resection, with an incidence of 6.7% following large EMR procedures 20 21 . Similar rates have been observed for ESD procedures, with post-procedural bleeding occurring in 0.5% to 9.6% of cases 12 22 23 . Post-procedural bleeding following endoscopic procedures is one of the most frequent reasons for endoscopic management of lower gastrointestinal bleeding, leading to a substantial cost burden for endoscopy services. Over the last two decades, several methods have been introduced into clinical practice to reduce risk of post-procedural bleeding, particularly after removal of larger lesions 1 . Mean size of lesions included in the study (52.28 ± 18.29 mm) presents a clinical challenge for closure via standard methods, such as clipping or suturing. Consequently, application of a simple hemostatic agent may emerge as a viable alternative strategy.

In our study involving 50 lesions, we applied HaemoCer PLUS powder after endoscopic removal using EMR or ESD techniques. We observed a 6% delayed bleeding rate, which is consistent with current literature reports 1 . Importantly, all instances of bleeding in our study occurred in lesions with a high estimated risk of delayed bleeding 20 . Of the three patients who experienced post-procedural bleeding, two had cecal lesions treated with the hot-snare technique and were on both antiplatelet and anticoagulant therapy.

Location in the proximal colon for EMR and the rectum for ESD 5 6 , lesion size greater than 30 mm, invasive cancer, and ongoing antithrombotic therapy are well-known risk factors for post-procedural bleeding 20 . Furthermore, in our study, proximal colonic lesions were not treated with clipping as per the study design, despite recent evidence emerging during the enrollment period showing that clip placement after large EMR in the proximal colon significantly reduces risk of post-procedural bleeding in patients on antithrombotic therapy 21 24 .

Antiplatelet therapy is a well-known risk factor for delayed bleeding after endoscopic resection 21 . In two cases in which bleeding complications occurred, the therapy could not be discontinued as recommended by European guidelines 18 due to cardiology recommendations. In addition, the hot-snare resection technique has been linked to a higher risk of bleeding compared with the cold-snare technique 2 25 .

In this study, post-procedural bleeding complications were categorized as mild for rectal ESD and moderate for proximal colon EMR according to the ASGE lexicon, and in all three cases, snare tip coagulation was applied as a prophylactic hemostatic method after resection 19 . None of the patients needed blood transfusions. For the two cases of cecal post-procedural bleeding, an endoscopic evaluation was conducted. In one case, an additional dose of HaemoCer PLUS was applied to the surface of the resected lesion with no active bleeding at the time of endoscopic evaluation. In the other case, active oozing was identified and treated with thermal ablation. In the patient with rectal bleeding, only a single dose of hemostatic powder was administered. The lesion, as measured in the pathological specimen, had a diameter of 85 mm. It is possible that application of the hemostatic agent in this case was not optimal.

Among the three patients who experienced post-procedural bleeding, two had underlying chronic liver disease, which should not be overlooked. In other studies, hemostatic powder has shown a comparable bleeding rate of 2.3% following ESD, similar to our observed rate of 5% (1 of 20). Hemostatic powder could be a valid option not only for the general population but also, in our opinion, for high-risk groups characterized by specific lesions and patient factors. Risk of bleeding is particularly high in patients on antithrombotic and antiplatelet therapy, and bleeding complications in these patients should not be underestimated due to their underlying cardiac comorbidities. Correct timing of medication suspension and resumption is crucial. If early resumption of these drugs is necessary, clot formation stimulation by hemostatic powder could be a beneficial mechanism.

In our sample, post-procedural bleeding occurred 24 hours after the procedure, and no bleeding cases were reported until 28 days after the endoscopy procedure. This may be a crucial factor for patients who need to resume antiplatelet or anticoagulant therapy 2 to 3 days after the procedure if the powder represents a protective factor during the phase of therapy resumption. Hemostatic powder was tested to reduce delayed bleeding complications, and the cost of this strategy for bleeding prevention was also analyzed 26 27 .

Clip closure has been shown to be cost-effective for high-risk bleeding lesions (with an assessed bleeding risk of at least 10%). Shah's study 28 showed that in the United States, delayed bleeding after colon resection can increase healthcare costs for patients by approximately $600. In the model proposed by the study, clipping methods were minimally costly in lesions with very high bleeding risk and reduced bleeding by more than 70%. To the best of our knowledge, the only available data comparing hemostatic powder or other endoscopic interventions are focused on comparing active bleeding entities.

A study compared hemostatic powder with standard endoscopic therapy for managing active gastrointestinal neoplasia 28 . The cost analysis, including rebleeding and readmission rates and associated costs, showed that hemostatic powder is a cost-saving option, especially for patients at high risk of rebleeding. One of the advantages of hemostatic powder is its simple administration, requiring no special expertise. In addition, no side effects have been reported. This pilot study shows promising results regarding the rate of delayed bleeding in patients undergoing EMR or ESD for large colonic lesions. The findings could be the basis for larger studies with control groups to further understand the mechanism of action and efficacy.

The study strengths include its multicenter and prospective design. This is the first experience with this new type of hemostatic powder in human subjects following large colonic polyp resection. Prior research on this agent has been exclusively confined to the surgical domain 29 30 .

This study findings, while promising, must be interpreted in context of several limitations that underscore clear directions for future research. First are the limited sample size without a priori power assessment and absence of a control group, which did not allow for obtaining solid data on efficacy. Furthermore, a significant knowledge gap remains regarding the agent's residence time on the post-resection ulcer bed.

Therefore, future research should also include a larger, controlled study adequately powered for subgroup analysis based on resection techniques (e.g., EMR vs. ESD) to account for their different intrinsic bleeding risks.

## Conclusions

In conclusion, HaemoCer PLUS appears to be a promising novel agent for reducing post-bleeding rates after large colonic resection. Lack of a learning curve linked to the simple application makes it highly suitable for widespread adoption. However, further studies, including a comprehensive cost-effectiveness analysis, are essential before its routine use can be recommended.

## References

[1] Ferlitsch M Hassan C Bisschops R Colorectal polypectomy and endoscopic mucosal resection: European Society of Gastrointestinal Endoscopy (ESGE) Guideline - Update 2024 Endoscopy 2024 56 516 545 10.1055/a-2304-3219 38670139

[2] O’Sullivan T Cronin O van Hattem WA Cold versus hot snare endoscopic mucosal resection for large (≥15 mm) flat non-pedunculated colorectal polyps: a randomised controlled trial Gut 2024 73 1823 1830 10.1136/gutjnl-2024-332807 38964854

[3] Pimentel-Nunes P Libânio D Bastiaansen BAJ Endoscopic submucosal dissection for superficial gastrointestinal lesions: European Society of Gastrointestinal Endoscopy (ESGE) Guideline - Update 2022 Endoscopy 2022 54 591 622 10.1055/a-1811-7025 35523224

[4] Shigita K Oka S Tanaka S Long-term outcomes after endoscopic submucosal dissection for superficial colorectal tumors Gastrointest Endosc 2017 85 546 553 10.1016/j.gie.2016.07.044 27475492

[5] Buddingh KT Herngreen T Haringsma J Location in the right hemi-colon is an independent risk factor for delayed post-polypectomy hemorrhage: a multi-center case-control study Am J Gastroenterol 2011 106 1119 1124 10.1038/ajg.2010.507 21266961

[6] Rutter MD Nickerson C Rees CJ Risk factors for adverse events related to polypectomy in the English Bowel Cancer Screening Programme Endoscopy 2014 46 90 97 10.1055/s-0033-1344987 24477363

[7] Russo P Barbeiro S Awadie H Management of colorectal laterally spreading tumors: a systematic review and meta-analysis Endosc Int Open 2019 7 E239 E259 10.1055/a-0732-487 30705959 PMC6353652

[8] Ortigão R Weigt J Afifi A Cold versus hot polypectomy/endoscopic mucosal resection-A review of current evidence United European Gastroenterol J 2021 9 938 946 10.1002/ueg2.12130 PMC8498395 34355525

[9] Spadaccini M Albéniz E Pohl H Prophylactic clipping after colorectal endoscopic resection prevents bleeding of large, proximal polyps: Meta-analysis of randomized trials Gastroenterology 2020 159 148 158 e11 32247023 10.1053/j.gastro.2020.03.051

[10] Albéniz E Fraile M Ibáñez B A scoring system to determine risk of delayed bleeding after endoscopic mucosal resection of large colorectal lesions Clin Gastroenterol Hepatol 2016 14 1140 1147 10.1016/j.cgh.2016.03.021 27033428

[11] Albéniz E Álvarez MA Espinós JC Clip closure after resection of large colorectal lesions with substantial risk of bleeding Gastroenterology 2019 157 1213 1221 e4 31362007 10.1053/j.gastro.2019.07.037

[12] Seo M Song EM Cho JW A risk-scoring model for the prediction of delayed bleeding after colorectal endoscopic submucosal dissection Gastrointest Endosc 2019 89 990 998 e2 30521794 10.1016/j.gie.2018.11.029

[13] ASGE Technology Committee Wong Kee Song L-M Banerjee S Emerging technologies for endoscopic hemostasis Gastrointest Endosc 2012 75 933 937 22445927 10.1016/j.gie.2012.01.024

[14] Burghuber CK Kandioler D Strobl S Standardized intraoperative application of an absorbable polysaccharide hemostatic powder to reduce the incidence of lymphocele after kidney transplantation - a prospective trial Transpl Int 2019 32 59 65 10.1111/tri.13329 30099769 PMC7380033

[15] Cahyadi O Bauder M Meier B Effectiveness of TC-325 (Hemospray) for treatment of diffuse or refractory upper gastrointestinal bleeding - a single center experience Endosc Int Open 2017 5 E1159 E1164 10.1055/s-0043-118794 29124127 PMC5677459

[16] Barkun AN Moosavi S Martel M Topical hemostatic agents: a systematic review with particular emphasis on endoscopic application in GI bleeding Gastrointest Endosc 2013 77 692 700 10.1016/j.gie.2013.01.020 23582528

[17] Shin J Cha B Park J-S Efficacy of a novel hemostatic adhesive powder in patients with upper gastrointestinal tumor bleeding BMC Gastroenterol 2021 21 40 33509102 10.1186/s12876-021-01611-0 PMC7842074

[18] Veitch AM Radaelli F Alikhan R Endoscopy in patients on antiplatelet or anticoagulant therapy: British Society of Gastroenterology (BSG) and European Society of Gastrointestinal Endoscopy (ESGE) guideline update Gut 2021 70 1611 1628 10.1136/gutjnl-2021-325184 34362780 PMC8355884

[19] Cotton PB Eisen GM Aabakken L A lexicon for endoscopic adverse events: report of an ASGE workshop Gastrointest Endosc 2010 71 446 454 10.1016/j.gie.2009.10.027 20189503

[20] Albéniz E Montori S Rodríguez de Santiago E Preventing postendoscopic mucosal resection bleeding of large nonpedunculated colorectal lesions Am J Gastroenterol 2022 117 1080 1088 35765907 10.14309/ajg.0000000000001819

[21] Forbes N Gupta S Frehlich L Clip closure to prevent adverse events after EMR of proximal large nonpedunculated colorectal polyps: meta-analysis of individual patient data from randomized controlled trials Gastrointest Endosc 2022 96 721 731 e2 35667388 10.1016/j.gie.2022.05.020

[22] Shigita K Oka S Tanaka S Long-term outcomes after endoscopic submucosal dissection for superficial colorectal tumors Gastrointest Endosc 2017 85 546 553 10.1016/j.gie.2016.07.044 27475492

[23] Ninomiya Y Oka S Tanaka S Risk of bleeding after endoscopic submucosal dissection for colorectal tumors in patients with continued use of low-dose aspirin J Gastroenterol 2015 50 1041 1046 25682173 10.1007/s00535-015-1053-4

[24] Turan AS Pohl H Matsumoto M The role of clips in preventing delayed bleeding after colorectal polyp resection: An individual patient data meta-analysis Clin Gastroenterol Hepatol 2022 20 362 371 e23 33991691 10.1016/j.cgh.2021.05.012

[25] Takeuchi Y Shichijo S Uedo N Safety and efficacy of cold versus hot snare polypectomy including colorectal polyps ≥1 cm in size Dig Endosc 2022 34 274 283 10.1111/den.14096 34324730

[26] Pohl H The cost of clipping-How much does price matter? Am J Gastroenterol 2021 116 276 277 33306507 10.14309/ajg.0000000000001088

[27] Albéniz E Enguita-Germán M Gimeno-García AZ The answer to "when to clip" after colorectal endoscopic mucosal resection based on a cost-effectiveness analysis Am J Gastroenterol 2021 116 311 318 33149001 10.14309/ajg.0000000000000943

[28] Shah ED Pohl H Rex DK Valuing innovative endoscopic techniques: prophylactic clip closure after endoscopic resection of large colon polyps Gastrointest Endosc 2020 91 1353 1360 10.1016/j.gie.2020.01.018 31962121

[29] Falcone V Krotka P Deutschmann C Use of polysaccharide hemostatic agent (HaemoCer) in breast cancer surgery to reduce postoperative complications: A randomised controlled trial Int Wound J 2023 20 925 934 10.1111/iwj.13939 36448255 PMC10031209

[30] Docimo G Filograna Pignatelli M Ferrandes S Role of absorbable polysaccharide hemostatic powder in the prevention of bleeding and wound events after thyroid surgery J Clin Med 2023 12 5684 37685750 10.3390/jcm12175684 PMC10488928

